# Platelet to lymphocyte ratio as an independent prognostic indicator for prostate cancer patients receiving androgen deprivation therapy

**DOI:** 10.1186/s12885-016-2363-5

**Published:** 2016-05-24

**Authors:** Yanqing Wang, Fan Xu, Jiahua Pan, Yinjie Zhu, Xiaoguang Shao, Jianjun Sha, Zezhou Wang, Yong Cai, Qiang Liu, Baijun Dong, Wei Xue, Yiran Huang

**Affiliations:** Department of Urology, Renji Hospital, School of Medicine, Shanghai Jiao Tong University, 1630 Dongfang Road, Pudong District, Shanghai, 200127 People’s Republic of China; School of Public Health, Shanghai Jiao Tong University, Shanghai, China; Department of Pathology, Renji Hospital, School of Medicine, Shanghai Jiao Tong University, Shanghai, China

**Keywords:** Prostate cancer, PLR, Prognostic factor, Survival

## Abstract

**Background:**

Platelet to Lymphocyte ratio (PLR) is thought to be associated with a worse outcome in multiple types of cancer. However, the prognostic significance of PLR has not been investigated in the prostate cancer (PCa) patients receiving hormonal therapy. The objective of this study was to determine the prognostic value of PLR in PCa patients treated with androgen deprivation therapy (ADT).

**Methods:**

Two-hundred-ninety prostate cancer patients who had undergone ADT as first-line therapy were retrospectively analyzed. The blood cell counts were performed at the time of diagnosis. PLR was calculated as the ratio of platelet count to lymphocyte count. Patients were categorized in two groups using a cut-off point of 117.58 as calculated by the receiver-operating curve analysis. Correlations between PLR and clinical characteristics were analyzed. Meanwhile, univariate and multivariate cox regression analyses were performed to determine the associations of PLR with progression-free survival (PFS), cancer-specific survival (CSS) and overall survival (OS). Prognostic accuracy was evaluated with the Harrell concordance index.

**Results:**

The differences of age, serum prostate-specific antigen (PSA) level, Gleason score, risk stratification and incidence of metastasis between low PLR group (<117.58) and high PLR group (≥117.58) were not statistically significant (*p* > 0.05). Multivariate analyses identified PLR as an independent prognostic factor for PFS (hazard ratio (HR) = 1.581, *p* = 0.013), CSS (HR = 1.768, *p* = 0.037) and OS (HR = 1.650, *p* = 0.044). The addition of PLR to the final model improved predictive accuracy (c-index: 0.747, 0.801 and 0.768) for PFS, CSS and OS compared with the clinicopathological base model (c-index: 0.730, 0.778 and 0.746), which included Gleason score and incidence of metastasis.

**Conclusions:**

PLR might play a significant role in the prognosis of PCa patients treated with ADT. Thus, we recommend adding PLR to traditional prognostic model to improve the predictive accuracy.

**Electronic supplementary material:**

The online version of this article (doi:10.1186/s12885-016-2363-5) contains supplementary material, which is available to authorized users.

## Background

Prostate cancer (PCa) is the most commonly diagnosed cancer and the second leading cause of cancer death in men in the United States [1]. Androgen deprivation therapy (ADT) is the mainstay of therapy for patients with locally advanced or metastatic PCa or patients with early-stage disease who are ineligible for local regional treatments due to health disparity [2].

A growing body of evidence suggests that inflammation might have a major role in the tumorigenesis and progression of PCa [3–5]. Low serum neutrophil count predicts a positive prostate biopsy [6]. The neutrophil to lymphocyte ratio (NLR) seems to represent an independent prognostic marker in patients with PCa [7]. Similarly, platelet to lymphocyte ratio (PLR) is also a systemic inflammation-based parameter. Numerous studies have revealed that high pretreatment PLR independently predicts poor prognosis in patients with tumors including gastric cancer [8], pancreatic cancer [9], ovarian cancer [10], colorectal cancer [11], non-small cell lung cancer [12], hepatocellular carcinoma [13], renal cell cancer [14], esophageal cancer [15]. Yuksel OH et al. [16] and Kaynar M et al. [17] reported that PLR could be used to distinguish benign prostatic hyperplasia and prostate cancer, in support of its diagnostic value. Langsenlehner T et al. [18] showed a significant association between PLR and prognosis of PCa patients who underwent radiation therapy. However, whether PLR plays an important role in the prognosis of PCa treated with ADT has not been reported.

Platelet and lymphocyte counts are routinely performed in most clinical laboratories worldwide, therefore we evaluated whether pretreatment PLR could predict the clinical outcome of PCa patients treated with ADT.

## Methods

### Study population

After obtaining approval from the Ethics Committee at the Renji Hospital, Shanghai Jiao Tong University School of Medicine, and informed consent from patients, medical records of 325 prostate cancer patients who had undergone ADT as first-line therapy at the Renji Hospital between January 2010 and December 2014 were retrospectively reviewed. We excluded patients with coagulation-related diseases, inflammatory diseases, autoimmune diseases, cerebrovascular diseases, other types of cancer, and those patients lost to follow-up. We finally assembled a cohort of 290 prostate cancer patients who had blood cell counts performed within 2 weeks before prostate biopsy.

### Clinical and pathological evaluation

Medical data on clinical characteristics including age, serum prostate-specific antigen (PSA) level and blood cell counts at diagnosis, clinical tumor stage, biopsy Gleason score and follow-up information were collected. The pathologic slides were re-reviewed by the urologic pathologists, and the Gleason scores were obtained from the original pathology reports. For clinical tumor stage, patients underwent pelvic Computed Tomography (CT) or Magnetic Resonance Imaging (MRI). Radionuclide bone scan was performed to determine whether there was bone metastasis. PCa patients were stratified into low-, intermediate-, and high-risk groups according to the EAU guidelines [19]: low-risk group, PSA < 10 μg/L and Gleason Score <7 and cT1c-2a; intermediate-risk group, PSA 10–20 μg/L or Gleason Score 7 or cT2b-2c; and high-risk group, PSA > 20 μg/L or Gleason Score 8–10 or > cT2c.

Eligible patients were treated with continuous ADT as first-line therapy, including castration and antiandrogen therapy. Castration involved surgical or medical castration by using a luteinizing hormone releasing hormone (LHRH) agonist, such as goserelin 3.6 mg, administered subcutaneously every month. Antiandrogen therapy was by bicalutamide tablets 50 mg per day orally or flutamide 3 times a day, 250 mg each time orally.

### Follow-up

Patients were followed for survival information every 3 months. Duration of the follow-up was assessed from the date of treatment until the last follow-up (June 2015) or death, which was defined as cancer-related or a different cause. Progression was defined as castration-resistance or death, and the castration-resistance was judged according to the EAU guidelines [20]. The median follow-up duration was 37.0 months (IQR, 24.0–50.3).

### Laboratory assays

Venous blood samples were collected before the prostate biopsy. Pre-biopsy platelet and lymphocyte counts were performed as part of routine clinical procedures to exclude coagulation disorders or presence of acute infection.

### Statistical analysis

The Wilcoxon Signed Rank test was used to interrogate the median with interquartile ranges (IQRs) between PLR and clinical characteristics, while chi-squared tests were used for categorical variables. The cut-off value for the continuous variable PLR was determined by applying a receiver operating characteristics curve analysis to test all possible cut-offs that would separate between patients’ survival and cancer-related death. The survival distributions, including progression-free survival (PFS), cancer-specific survival (CSS) and overall survival (OS) were estimated by the Kaplan–Meier method and compared by a log-rank test, and subgroup analyses were taken according to Gleason score and incidence of metastasis. PFS was calculated from the date of prostate biopsy to the date of disease progression or the time of the last follow-up. The effect of PLR on PFS, CSS and OS were examined using cox proportional hazard regression models. All variables including PLR with a *p* value <0.05 on univariate analyses were entered into multivariate stepwise cox regression analyses. Hazard ratio (HR) and 95 % confidence interval (CI) were computed. To examine whether PLR can provide additional prognostic power when combined with basic clinical variables, we built predictive model and calculated the c-index by integrating clinical variables with PLR using the R package “survival.” For each core set, we randomly extracted 20 % samples as the test set to generate a c-index, and the above procedure was repeated 100 times to generate 100 c-indexes. Then, we used the Wilcoxon signed rank test to calculate the *p* value. All tests were two-sided. Differences were considered to be statistically significant if *p* < 0.05. Statistical analysis was carried out using SPSS, version 19.0.

## Results

### Clinical characteristics

The clinical characteristics of the patients are detailed in Table 1. The median age of the patients was 75 years old (IQR, 67–79).

**Table 1:** Clinical characteristics of prostate cancer patients treated with ADT (*n* = 290)

Parameters	No. of patients (%)
Age (median, interquartile range), years	75 (67–79)
PSA (median, interquartile range), μg/L	100.00 (31.13–198.50)
Gleason Score	
< 7	36 (12.41)
7	108 (37.24)
> 7	146 (50.35)
Metastasis	
No	159 (54.83)
Yes	131 (45.17)
Risk Stratification	
Low	1 (0.34)
Intermediate	27 (9.31)
High	262 (90.35)
Platelet (median, interquartile range), 109/L	181.50 (145.00–215.25)
Lymphocyte (median, interquartile range), 109/L	1.54 (1.21–1.89)
PLR	117.46 (87.26–154.57)
Progression-free survival	126 (43.45)
Cancer-specific survival	60 (20.69)
Overall survival	70 (24.14)
Follow-up time (months)	37.00 (24.00–50.30)

*Abbreviations*: *PSA* prostate-specific antigen

### Association between PLR and clinical and pathological characteristics

Based on ROC curve for survival analysis (CSS), the best cut-off value for PLR was 117.58, and all patients were divided into either low PLR group (*n* = 146, 50.34 %) or high PLR group (*n* = 144, 49.66 %). The differences of age, serum PSA level, Gleason score, risk stratification and incidence of metastasis between low PLR group and high PLR group were not significant (*p* > 0.05). (Table 2).

**Table 2:** Clinical characteristics of prostate cancer patients according to PLR

Parameters	PLR		*P*-value
	<117.58 (*n* = 146 50.34 %)	≥117.58 (*n* = 144 49.66 %)	
Age (median, interquartile range), years	76 (66–79)	75 (68–80)	0.632
PSA (median, interquartile range), μg/L	100.00 (28.30–170.00)	100.00 (33.05–213.00)	0.625
Gleason Score (≤7/>7)	76/70	68/76	0.410
Metastasis (no/yes)	87/59	72/72	0.100
Risk Stratification (low intermediate/high)	15/131	13/131	0.719
Platelet (median, interquartile range), 109/L	159.50 (134.00–198.00)	201.00 (167.50–238.50)	<0.001
Lymphocyte (median, interquartile range), 109/L	1.84 (1.57–2.21)	1.24 (1.02–1.51)	<0.001

*Abbreviations*: *PSA*, prostate-specific antigen

### Association between PLR and prognosis of PCa

The median follow-up duration was 37.0 months, out of 290 patients with usable follow-up information, 126 (43.45 %) patients experienced disease progression, and 70 (24.14 %) patients died, including 60 (20.69 %) patients died of PCa at the end of the last follow-up.

**Fig. 1:**
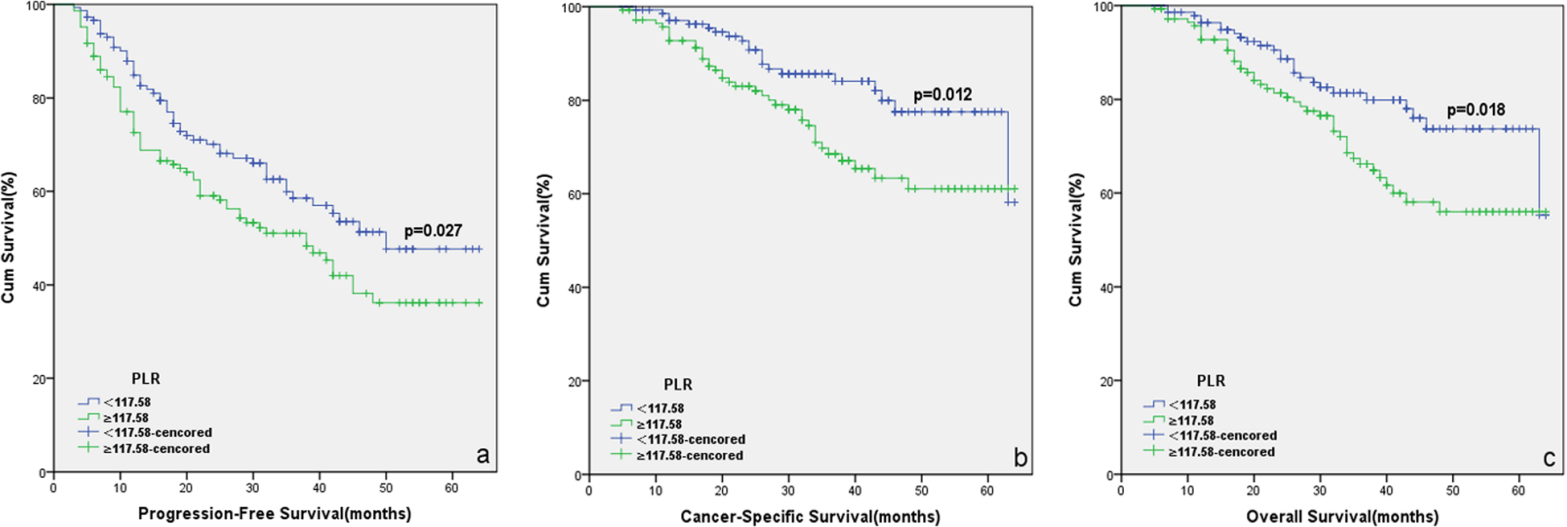
Kaplan-Meier curves for survival of prostate cancer patients according to PLR. **a** Progression-free survival (PFS), **b** Cancer-specific survival (CSS) and **c** Overall survival (OS)

**Fig. 2:**
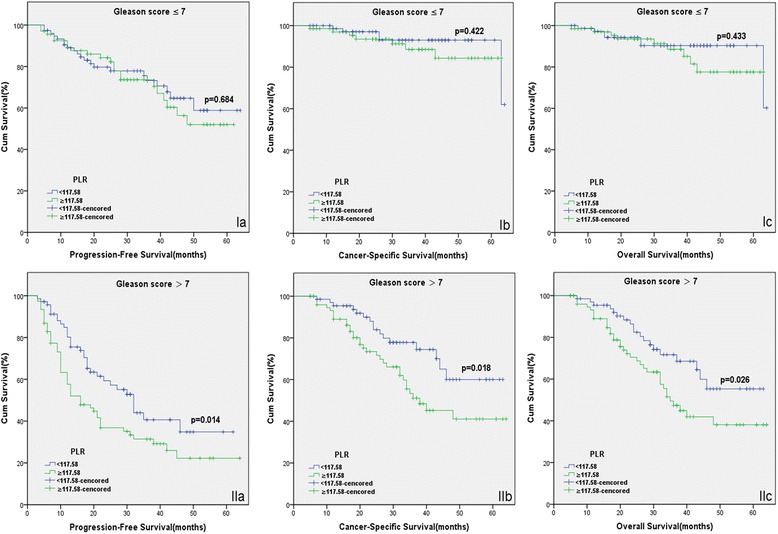
Kaplan-Meier survival curves stratified by PLR in prostate cancer patients with Gleason score ≤7 (*I*) and Gleason score >7 (*II*). **a** Progression-free survival (PFS), **b** Cancer-specific survival (CSS) and **c** Overall survival (OS)

**Fig. 3:**
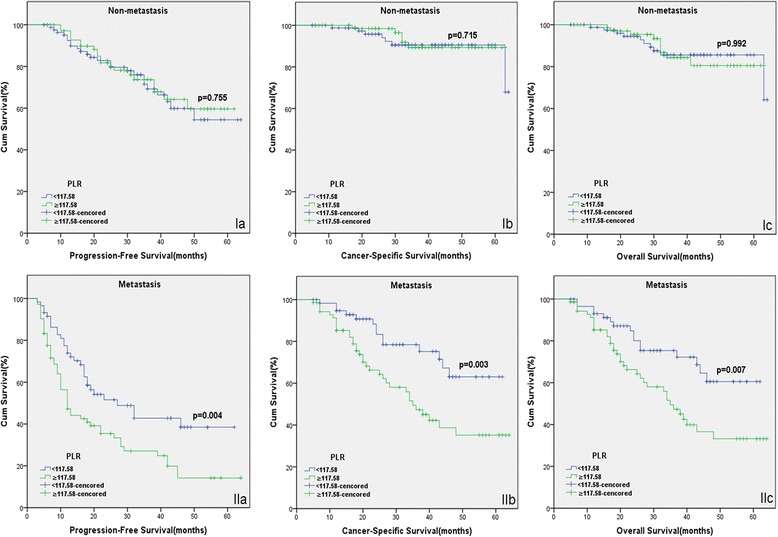
Kaplan-Meier survival curves stratified by PLR in prostate cancer patients with non-metastasis (*I*) and metastasis (*II*). **a** Progression-free survival (PFS), **b** Cancer-specific survival (CSS) and **c** Overall survival (OS)

The patients with high PLR had a significantly worse survival than those with low PLR with regard to PFS, CSS and OS (Log-rank test, each *P* < 0.05, Fig. 1). As shown in Figs. 2 and 3, in the subgroup of patients with Gleason score >7 or bone metastasis, high PLR group predicted the worse PFS, CSS and OS (Log-rank test, each *P* < 0.01). However in the subgroup of Gleason score ≤7 or non-metastasis, the prognostic differences of clinical outcome were not significant between high PLR group and low PLR group (Log-rank test, each *P* > 0.05). Univariate and multivariate cox regression analyses (stepwise analysis) of the factors influencing PFS, CSS and OS were presented in Tables 3 and 4. Univariate analyses demonstrated that serum PSA level, Gleason score, incidence of metastasis, PLR were significant predictors for PFS, CSS and OS (each *P* < 0.05), but age and risk stratification were significant predictors for PFS, not for CSS and OS. In the multivariate analyses, we identified that age, Gleason score, incidence of metastasis and PLR were independent prognostic factors for PFS, while Gleason score, incidence of metastasis and PLR were independent prognostic factors for CSS and OS. The HRs of PLR were 1.581 (95 % CI 1.100–2.272) for PFS, 1.768 (95 % CI 1.036–3.015) for CSS and 1.650 (95 % CI 1.013–2.687) for OS, respectively.

**Table 3:** Univariate analyses of various clinical parameters in prostate cancer patients

Parameters	Progression-Free survival		Cancer-Specific survival		Overall survival	
	HR (95 % CI)	*P*-value	HR (95 % CI)	*P*-value	HR (95 % CI)	*P*-value
Age (years)	0.958 (0.938–0.981)	<0.001	0.993 (0.962–1.024)	0.641	1.005 (0.975–1.035)	0.749
PSA (μg/L)	1.003 (1.002–1.004)	<0.001	1.003 (1.001–1.005)	0.002	1.002 (1.000–1.004)	0.029
Gleason Score		<0.001		<0.001		<0.001
≤ 7	1		1		1	
> 7	2.791 (1.921–4.055)		4.710 (2.499–8.878)		4.998 (2.286–6.993)	
Metastasis		<0.001		<0.001		<0.001
No	1		1		1	
Yes	3.447 (2.385–4.984)		5.893 (3.128–11.102)		3.951 (2.331–6.696)	
Risk Stratification		0.005		0.058		0.111
Lowintermediate	1		1		1	
High	4.128 (1.524–11.182)		6.751 (0.935–48.730)		2.560 (0.805–8.140)	
Platelet (10^9^/L)		0.053		0.157		0.121
< 190.50	1		1		1	
≥ 190.50	1.414 (0.995–2.003)		1.442 (0.869–2.394)		1.450 (0.907–2.318)	
Lymphocyte (10^9^/L)	0.866 (0.632–1.185)	0.367	0.724 (0.454–1.153)	0.174	0.775 (0.506–1.188)	0.242
PLR		0.029		0.014		0.020
< 117.58	1		1		1	
≥ 117.58	1.480 (1.040–2.107)		1.948 (1.145–3.312)		1.781 (1.096–2.894)	

*Abbreviations*: *HR* hazard ratio, *CI* confidence interval, *PSA* prostate-specific antigen

**Table 4:** Multivariate analyses of various clinical parameters in prostate cancer patients

Parameters	Progression-Free survival		Cancer-Specific survival		Overall survival	
	HR (95 % CI)	*P*-value	HR (95 % CI)	*P*-value	HR (95 % CI)	*P*-value
Age (years)	0.975 (0.955–0.996)	0.023		—		—
PSA (μg/L)		0.093		0.916		0.480
Gleason Score		<0.001		<0.001		<0.001
≤ 7	1		1		1	
> 7	2.136 (1.454–3.137)		3.378 (1.776–6.428)		3.086 (1.747–5.453)	
Metastasis		<0.001		<0.001		<0.001
No	1		1		1	
Yes	2.830 (1.939–4.131)		4.505 (2.367–8.573)		3.080 (1.799–5.274)	
PLR		0.013		0.037		0.044
< 117.58	1		1		1	
≥ 117.58	1.581 (1.100–2.272)		1.768 (1.036–3.015)		1.650 (1.013–2.687)	

*Abbreviations*: *HR* hazard ratio, *CI* confidence interval, *PSA* prostate-specific antigen

The predictive accuracy was calculated with and without the inclusion of PLR. With the base model, including the traditional predictor variables of Gleason score and incidence of metastasis, predictive accuracy for PFS, CSS and OS was 73.0 % (IQR, 71.1–77.1 %), 77.8 % (IQR, 73.1–81.9 %) and 74.6 % (IQR, 71.8–77.7 %). Our new integrated model with the addition of PLR, predictive accuracy for PFS, CSS and OS was 74.7 % (IQR, 72.3–77.8 %), 80.1 % (IQR, 76.7–84.2 %) and 76.8 % (IQR, 73.7–79.9 %), respectively. Notably, our integrated model showed statistically significantly improved predictive power compared to the base model (each *p* < 0.001).

## Discussion

Despite recent progress in the identification of genetic and molecular alterations in prostate cancer (PCa), the routine prognostic risk assessment of PCa patients currently relies on traditional clinicopathological prognostic factors, including Gleason score, clinical tumor stage, and serum PSA level at the time of diagnosis, which are used for stratify the patients into the low-, intermediate-, or high-risk group [19]. The predictive accuracy of this traditional prognostic model need be further improved by the incorporation of novel prognostic biomarkers.

The platelet and lymphocyte counts are routinely measured blood-based parameters. In this large cohort of PCa patients treated with androgen deprivation therapy (ADT), we found that pretreatment high platelet to lymphocyte ratio (PLR) was associated with increased risk for disease recurrence, cancer specific mortality and all-cause mortality. These findings remained significant after adjusting for clinicopathological features, indicating an independent association of high pretreatment PLR with adverse outcomes.

An exact explanation for this observation remains unclear and is yet to be elucidated. The PLR represents a marker of inflammation. A high PLR reflects both an elevated platelet dependent pro-tumor reaction and a decreased lymphocyte mediated anti-tumor immune response, which may both contribute to cancer progression and poor outcome. Platelets have been shown to promote cancer growth and metastasis. For instance, Boucharaba A et al. [21] showed platelet-derived lysophosphatidic acid was critical for bone metastasis formation in breast cancer. Dashevsky O et al. [22] demonstrated platelet-derived microparticles promoted invasiveness of PCa cells via upregulation of MMP-2 production. Zheng S et al. [23] found, in PCa, fibrinogen help platelets to adhere to tumor cells, and platelets in turn promoted more fibrinogen to aggregate around tumor cells by forming thrombin, and thus protected tumor cells from natural killer cell cytotoxicity, which was mediated by β3-integrins. There is considerable evidence that lymphocytes represent the cellular basis of cancer immunosurveillance, which inhibit tumor cell proliferation and metastasization [24]. Huang SH et al. [25] showed pretreatment high circulating lymphocytes could predict better recurrence-free survival and marginally better overall survival in HPV^+^ oropharyngeal cancer patients. Adams S et al. [26] confirmed tumor stromal lymphocytic infiltration constituted a robust prognostic factor in triple-negative breast cancers from two phase III randomized adjuvant breast cancer trials. However, the differences of tumor features including age, serum PSA level, Gleason score, risk stratification and incidence of metastasis between low PLR group (<117.58) and high PLR group (≥117.58) were not significant in our study.

To date, adverse clinical outcomes of many cancers have been associated with an elevated PLR [8–15]. Zhou X et al. [27] performed a meta-analysis on the prognostic value of PLR in various cancers, and found that elevated PLR was a negative predictor for OS with HR of 1.60 (95 % CI 1.35–1.90, *P* < 0.001). Similarly, Templeton AJ et al. [28] conducted a systematic review and showed that a high PLR was associated with worse OS in various solid tumors.

In contrast to the well-known prognostic value of PLR in other malignancies, its value in PCa is poorly studied. Only two studies exist regarding the association of PLR and PCa outcomes. In 384 patients treated with 3D conformal radiotherapy from 1999 to 2007, Langsenlehner T et al. [18] noted an increased PLR (≥190) was a significant prognostic factor for poor distant metastases-free survival, cancer-specific survival and overall survival. Importantly, intergroup inconsistent neo-adjuvant therapy and potential confounding factors like inflammatory diseases may affect their study results. In 2015, Li F et al. [29] evaluated the relationship between PLR ≥150 and all cause mortality in 103 PCa patients, and found that PLR was an independent risk factor of 3-year mortality, However, their analysis was limited by its relatively small patient population and intergroup inconsistent therapy.

To the best of our knowledge, our analysis is the first study of PLR on the prognosis of PCa treated with ADT, and strikingly our results showed that PLR was an independent prognostic factor for PFS, CSS and OS. Comparing to previous studies, we made a subgroup analysis and built a new integrated prognostic model. In subgroup analysis, we found that high PLR could predict poor prognosis in the subgroup of patients with Gleason score >7 or bone metastasis. In the subgroup of Gleason score ≤7 or non-metastasis, however, PLR was not statistically significantly associated with prognosis, probably because the percentages of patients who reached the endpoints (progression, cancer-related death and overall death) in this subgroup were too small. With the traditional prognostic base model, which includes only Gleason score and incidence of metastasis, the predictive accuracy was 73.0, 77.8 and 74.6 % for PFS, CSS and OS, respectively. The predictive accuracy was clearly improved by the addition of PLR (74.7, 80.1 and 76.8 %).

There are some limitations to our current study. First, this was a retrospective investigation. Despite the strict enrollment criteria applied, we were unable to completely exclude conditions that might cause inflammatory changes in PCa. Second, the patient data were collected from a single institution. Our results need to be validated by prospective research and patient data from multiple medical centers.

## Conclusion

PLR might be a novel prognostic marker in predicting the clinical outcome for PCa patients treated with ADT. Thus, we recommend adding PLR to traditional prognostic model to improve the predictive accuracy.

## Additional file

Additional file 1: Original data of 290 prostate cancer patients receiving androgen deprivation therapy. (XLS 70 kb)

## Abbreviations

ADT: androgen deprivation therapy
CI: confidence interval
CSS: cancer-specific survival
HR: hazard ratio
IQR: interquartile range
NLR: neutrophil to lymphocyte ratio
OS: overall survival
PCa: prostate cancer
PFS: progression-free survival
PLR: Platelet to Lymphocyte ratio
PSA: prostate-specific antigen
